# Identification of a novel matrix metalloproteinases-related prognostic signature in hepatocellular carcinoma

**DOI:** 10.18632/aging.205832

**Published:** 2024-05-16

**Authors:** Xingxing Yuan, Liuxin Yang, Jiawei Gao, Xu Mao, Yali Zhang, Wei Yuan

**Affiliations:** 1 Department of Gastroenterology, Heilongjiang Academy of Traditional Chinese Medicine, Harbin, China; 2 Heilongjiang University of Chinese Medicine, Harbin, China; 3 Zhang Yali Famous Traditional Chinese Medicine Expert Studio, Harbin, China; 4 Department of Hepatology, The First Affiliated Hospital of Hunan University of Traditional Chinese Medicine, Changsha, China

**Keywords:** prognostic signature, hepatocellular carcinoma, matrix metalloproteinases, bioinformatic analysis, tumor microenvironment

## Abstract

Background: Hepatocellular carcinoma (HCC) is the most common primary liver cancer worldwide. Cancer cells’ local infiltration, proliferation, and spread are mainly influenced by the protein hydrolyzing function of different matrix metalloproteinases (MMPs). However, no study has determined the relationship between MMPs and prognostic prediction in HCC.

Methods: Expression profiles of mRNA and MMPs-related genes were obtained from publicly available databases. Cox regression and LASSO Cox regression analysis were used to identify and predict MMPs-related prognostic signature and construct predictive models for overall survival (OS). A nomogram was used to validate the accuracy of the prediction model. Drug prediction was performed using the Genomics of Drug Sensitivity in Cancer (GDSC) dataset, and single-cell clustering analysis was performed to further understand the significance of the MMPs-related signature.

Results: A MMPs-related prognostic signature (including RNPEPL1, ADAM15, ADAM18, ADAMTS5, CAD, YME1L1, AMZ2, PSMD14, and COPS6) was identified. Using the median value, HCC patients in the high-risk group showed worse OS than those in the low-risk group. Immune microenvironment analysis showed that patients in the high-risk group had higher levels of M0 and M2 macrophages. Drug sensitivity analysis revealed that the IC_50_ values of sorafenib, cisplatin, and cytarabine were higher in the high-risk group. Finally, the single-cell cluster analysis results showed that YME1L1 and COPS6 were the major genes expressed in the monocyte cluster.

Conclusions: A novel MMPs-related signature can be used to predict the prognosis of HCC. The findings of this research could potentially impact the predictability of the prognosis and treatment of HCC.

## INTRODUCTION

According to estimates by the World Health Organization (WHO), hepatocellular carcinoma (HCC) is the main reason behind cancer deaths worldwide, which accounts for more than 90% of primary liver cancers and is the fifth most commonly occurring malignancy [1, 2]. Patients with HCC commonly exhibit underlying liver disease, and various factors that increase the likelihood of developing HCC have been established, including viral infection with hepatitis C virus (HCV), viral infection with hepatitis B virus (HBV), cirrhosis, metabolic syndrome, alcoholism, as well as the consumption of aflatoxin B1 and smoking [3]. Despite notable progress in the management of HCC, encompassing surgical procedures like resection and liver transplantation, localized therapies such as ablation, transcatheter arterial chemoembolization, and transcatheter arterial radiation embolization, as well as systemic treatments, the outlook for HCC remains unsatisfactory. The estimated overall survival rate after five years is below 20% due to most patients being diagnosed at intermediate or advanced stages of the disease [4, 5]. The use of biomarkers is crucial in diagnosing diseases early, predicting prognosis, and optimizing treatment strategies, which leads to improved patient survival [6]. The high heterogeneity of HCC, coupled with the complex etiologic factors, makes prognostic prediction challenging. Therefore, it is essential to identify new prognostic models.

Solid tumors are complex structures composed of cancer cells surrounded by a vascularized, dynamic tumor stroma containing a variety of nonmalignant cells, such as myeloid cells and fibroblasts, which play an essential role in angiogenesis, cell motility, and extracellular matrix (ECM) remodeling [7]. Cancerous cells’ local infiltration, proliferation, and spread are mainly influenced by the proteolytic function of different matrix metalloproteinases (MMPs). These enzymes promote the activation of the immunity cells and facilitate the process of growth, movement, invasion, metastasis, and angiogenesis by breaking down components of the ECM and releasing growth factors, cytokines, or their receptors that bind to the cell surface [8]. Although multiple studies have verified that MMPs are significantly increased in nearly all types of certain types of cancer in humans, including bladder cancer and HCC, and their expression is commonly linked to unfavorable survival outcomes [9–13]. Nevertheless, the complete understanding of MMPs-related genes in the prognosis of HCC remains elusive.

In this study, patient data and mRNA expression data of HCC samples were collected from the TCGA database. Then, an MMPs-related prognostic signature was identified, and validation of this signature was conducted using cohorts from TCGA-LIHC and the ICGC-LIRI-JP. Additionally, we verified the cellular distribution and expression of these predictive genes using single-cell clustering analysis. This finding enhanced our understanding of the predictive model related to MMPs in HCC and contributed to the identification of possible novel therapeutic targets for HCC.

## MATERIALS AND METHODS

### Datasets

**Figure 1:**
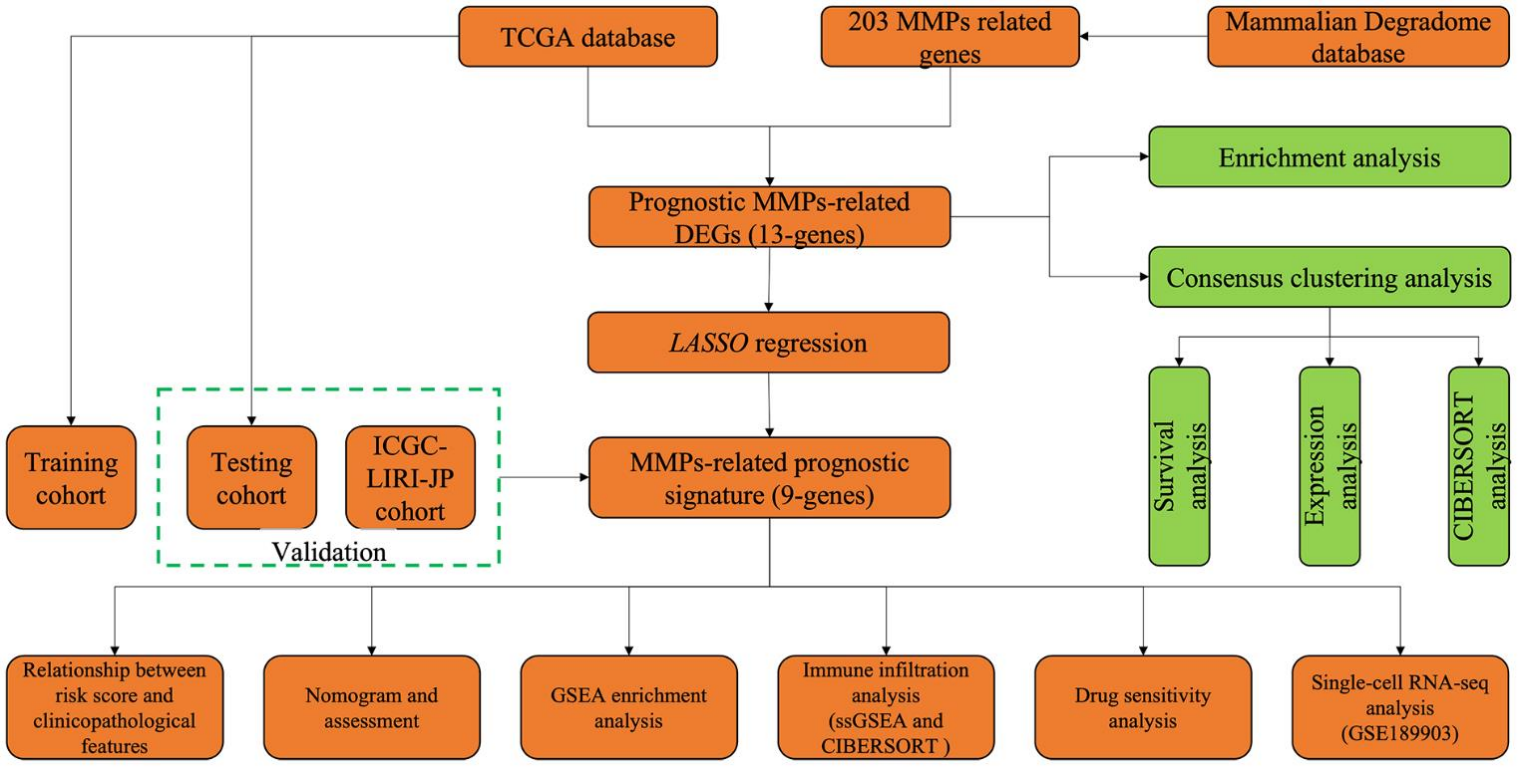
Flowchart for bioinformatics analysis in this study.

The database Mammalian Degradome (http://degradome.uniovi.es/dindex.html) was used to look for MMPs-related genes. RNA sequencing profiles and patient information for 374 HCC samples were downloaded from The Cancer Genome Atlas (TCGA) database (https://portal.gdc.cancer.gov/). In addition, 231 HCC patients from the International Cancer Genome Consortium for the Study of Liver Cancer in Japan (ICGC-LIRI-JP) dataset and their corresponding clinical characteristics were obtained from the ICGC database (https://dcc.icgc.org/). Furthermore, a single-cell sequencing set of HCC (GSE189903) was downloaded from the GEO database (https://www.ncbi.nlm.nih.gov/geo/). The acquisition of all publicly available data adheres to the publication and database access policies for the mentioned databases. A flowchart for bioinformatics analysis of publicly available datasets from the TCGA, ICGC, and GEO databases is shown in Figure 1.

### Identification of prognostic MMPs-related DEGs

A univariate Cox regression analysis was performed to assess the predictive potential of MMPs-related genes and identify the association between MMPs-related differentially expressed genes (DEGs) and survival status in the TCGA-LIHC cohort.

### Consensus clustering of prognostic MMPs-related DEGs

Clustering analysis of the expression patterns of survival-related MMP genes to explore the association between their expression and survival status was performed using the “ConsensuClusterPlus” package. One hundred replicates were performed with a pltem value of 0.8 to verify the stability of the subtypes. The clustering effect was best when the k-value was set to 2. Kaplan-Meier analysis was performed using the “survminer” package to estimate the differences in overall survival (OS) and progression-free survival (PFS) between different clusters. A log-rank test was performed to assess differences in survival. The above survival-related genes between the two clusters were analyzed using the “limma” package in R.

### Construction and validation of MMPs-related prognostic signature

For modeling purposes, unnecessary genes were removed by using LASSO Cox regression. The risk score was then calculated using a formula derived from the correlation coefficient and gene expression value obtained from the multifactorial Cox regression analysis. The formula was then applied to determine the scores, which were then used to categorize patients based on the median into high-risk and low-risk groups.

Subsequently, the TCGA cohort was assigned as a training cohort and a testing cohort, with the testing cohort serving as an internal validation cohort and the ICGC as an external validation cohort, both used to validate the predictive model.

To investigate the gene expression distribution in the predictive model, two separate analyses, namely t-distributed stochastic neighbor embedding (t-SNE) and principal component analysis (PCA), were undertaken to assess positioning for two distinct risk groups. We evaluated the OS between training and the testing cohorts by employing Kaplan-Meier analysis and visualized the results using the “survminer” package. The AUC was calculated at 1-, 3-, and 5-year intervals using the “time ROC” package to assess the accuracy of the predictive model.

### Risk score independent prognostic analysis

Univariate and multivariate Cox regression analyses assessed the relationship between risk factors and prognosis. Forest maps were plotted to show the independent predictive value of the risk score. A nomogram was created using the risk score and other clinical indicators to predict 1-, 3- and 5-year OS of HCC patients. To further evaluate the discriminative and predictive ability of the nomogram, we also calculated the concordance index (C-index). The C index values varied from 0.5 to 1.0, whereby a higher C index value indicates a higher discriminative ability of the prediction model [14].

### Analysis of functional enrichment

The analyses of Gene Ontology (GO) and the Kyoto Encyclopedia of Gene and Genomes (KEGG) were accomplished using the “clusterProfiler” package. Furthermore, we used Gene Set Enrichment Analysis (GSEA) to determine the significance of the MMPs-related prognostic signature in differentiating between the low and high-risk groups.

### Evaluation of the infiltration of immune cells

We employed single-sample Gene Set Enrichment Analysis (ssGSEA) as well as Cell-type Identification By Estimating Relative Subsets Of RNA Transcripts (CIBERSORT) to assess the magnitude with which the infiltration of immune cells was observed in different clusters and groups of the immune analysis. Gene set for ssGSEA was acquired via previous study [15]. The number of iterations of CIBERSORT was set to 1000. We excluded samples for which the P-value was less than 0.05. Additionally, the correlation between the immune cell profiles and the MMPs-related prognostic signature was examined using Spearman’s correlation. Based on the TCGA-LIHC profile of solid tumor expression, six immune subtypes (immune C1-C6) were identified, including wound healing, IFN-γ dominant, inflammatory, lymphocyte depleted, immunologically quiet, and TGF-beta dominant [16]. A two-way analysis of variance (ANOVA) was used to examine the correlation between the risk score and immune subtypes.

### Analysis of drug sensitivity

Genomics of Drug Sensitivity in Cancer (GDSC, https://www.cancerrxgene.org/) is an accessible dataset that provides comprehensive data regarding the sensitivity of cancer cells to drugs as well as drug-response molecular markers [17]. By employing the package of “oncopredict”, we distinguished the responsiveness to different groups of medications, enabling the assessment of 198 therapeutic compounds. The sensitivity scores were then evaluated to determine the predicted IC_50_ of all drugs in HCC patients.

### Profiling of RNA-seq at the single-cell level

The R package “Seurat” was used for unsupervised clustering of single cells from cancer or normal samples, and “Seurat” for annotation. Two clustering methods for dimensionality reduction, tSNE and Uniform Manifold Approximation and Projection (UMAP) were used in this study. The expression of the MMPs-related signature was visualized using the “VlnPlot” function of the “Seurat” R package.

### Statistics analysis

All statistical analyses were performed using R software (version 4.2.3). Continuous variables were tested using the student t-test, while categorical variables were tested using the chi-squared test. A *P*-value < 0.05 was considered significant.

## RESULTS

### Identification of prognostic MMPs-related DEGs

**Figure 2:**
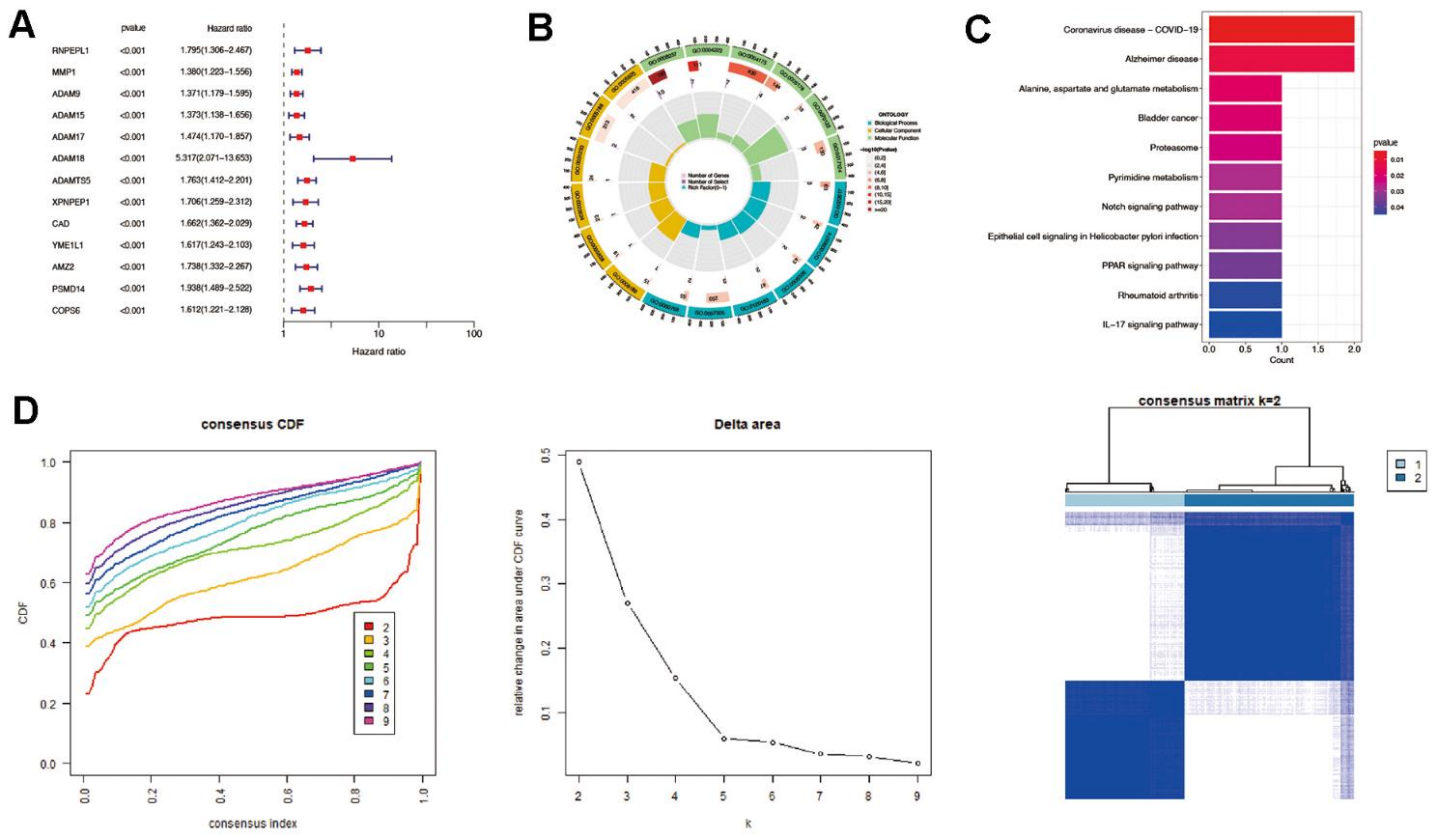
**Identification of prognostic MMPs-related DEGs in hepatocellular carcinoma.** (**A**) Prognostic MMPs-related DEGs are identified from MMPs-related genes through univariate Cox regression analysis. (**B**) GO enrichment analysis of prognostic MMPs-related DEGs, including biological processes (BP), molecular functions (MF), and cellular components (CC). (**C**) KEGG enrichment analysis of prognostic MMPs-related DEGs. (**D**) HCC patients were divided into two clusters according to the consensus clustering matrix (k = 2).

This study obtained 203 well-defined MMPs-related genes (Supplementary Table 1). A total of 13 prognostic MMPs-related DEGs significantly correlated with OS were identified from TCGA-LIHC cohort by univariate Cox regression analysis based on the criterion of FDR<0.005, including RNPEPL1, MMP1, ADAM9, ADAM15, ADAM17, ADAM18, ADAMTS5, XPNPEP1, CAD, YME1L1, AMZ2, PSMD14, and COPS6. (Figure 2A) Afterward, functional enrichment, including GO and KEGG analysis, was performed. The results of GO analysis indicated that the prognostic MMPs-related DEGs were mainly involved in extracellular matrix degradation, collagen catabolic process, membrane protein ectodomain proteolysis, membrane protein proteolysis, and extracellular matrix organisation (Figure 2B). The KEGG analysis result also suggested that the prognostic MMPs-related DEGs were involved in alanine, aspartate, and glutamate metabolism, Notch signaling pathway, PAR signaling pathway, and IL-17 signaling pathway (Figure 2C).

Consensus clustering analysis was then performed based on the expression levels of prognostic MMPs-related DEGs. HCC patients could be divided into the highest intra-group correlation and the lowest inter-group correlation (Figure 2D).

**Figure 3:**
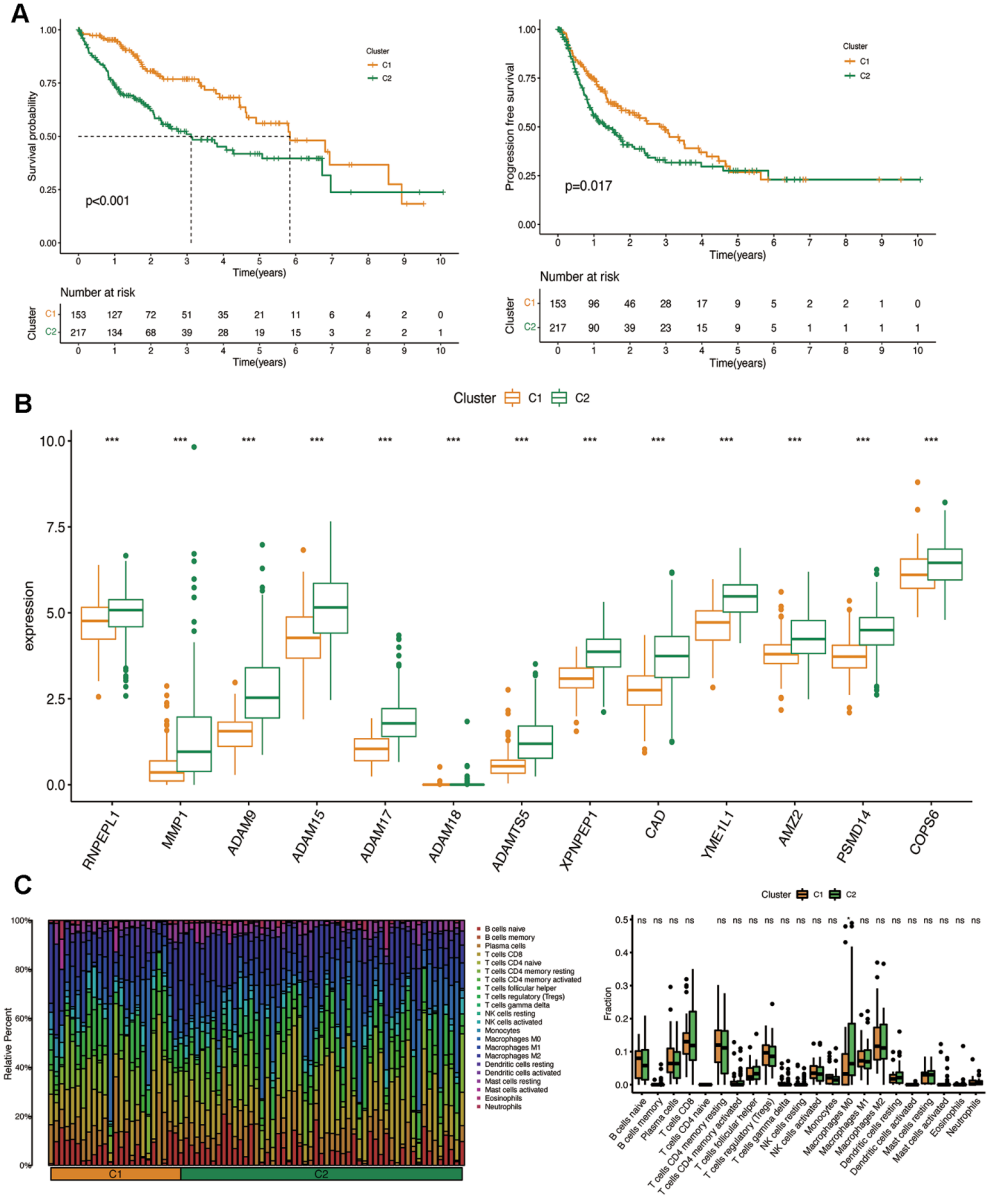
**Clinicopathological characteristics of the two clusters classified by prognostic MMPs-related DEGs.** (**A**) Kaplan–Meier curves for the OS and PFS in the two clusters. (**B**) Comparison of the expression of survival-related MMPs genes in the two clusters. (**C**) CIBERSORT analysis of infiltrating immune cells in the two clusters.

The OS and FPS rates of the two clusters were evaluated, revealing that cluster 1 demonstrated a significantly superior survival status in comparison to cluster 2 (*P*<0.001) (Figure 3A). The levels of prognostic MMPs-related DEGs were also evaluated in both clusters, and cluster 1 showed lower expression of these genes compared to cluster 2 (*P*=0.017) (Figure 3B). CIBERSORT was used to determine if the harmful effects of prognostic MMPs-related DEGs in HCC resulted from the infiltration of immune cells. This analysis revealed 22 different immune cell profiles from Cluster 1 and Cluster 2. According to the CIBERSORT analysis, the number of M0 macrophages in cluster 2 showed a substantial decrease when compared with cluster 1 (Figure 3C).

### Construction and validation of the MMPs-related prognostic signature

**Figure 4:**
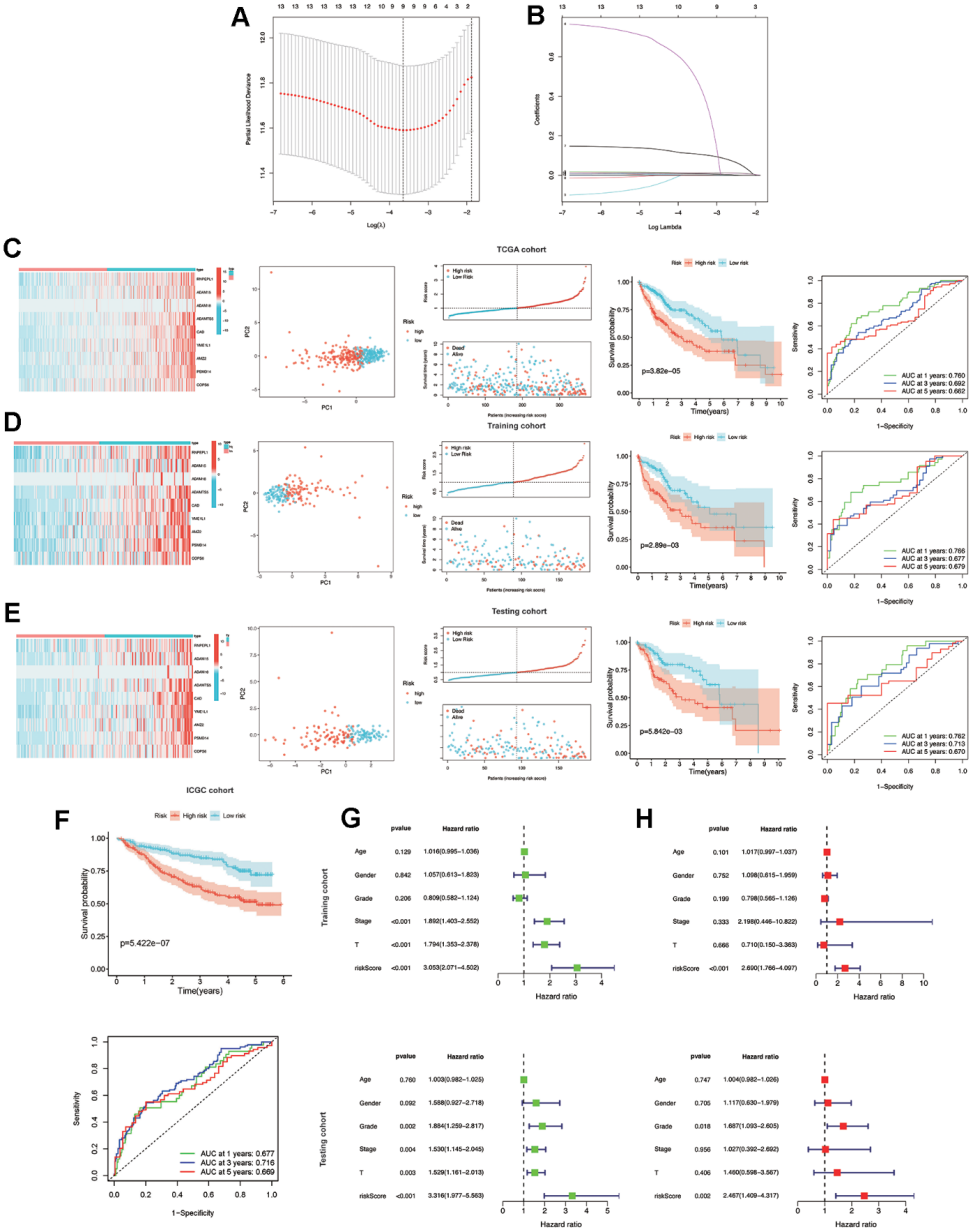
**Construction and validation of the MMPs-related prognostic signature.** (**A**) LASSO regression with tenfold cross-validation found nine prognostic genes using the minimum λ. (**B**) LASSO coefficient profiles of nine prognostic genes of HCC. (**C**, **D**) Heatmap, PCA plot, distribution, survival status, Kaplan-Meier curves for OS and ROC curves demonstrated the predictive efficiency of the risk score in entire, training, and testing cohorts. (**E**) Validation of the MMPs-related prognostic signature in the training cohort of TCGA-LIHC. (**F**) The validation of the MMPs-related prognostic signature in the ICGC-LIRI-JP cohort. (**G**) Univariate analysis of risk score and clinicopathological characteristics in the TCGA-LIHC training and testing cohorts. (**H**) Multivariate analysis of risk score and clinicopathological characteristics in the TCGA-LIHC training and testing cohorts.

Subsequently, a LASSO Cox regression analysis was carried out on prognostic MMPs-related DEGs, using the one standard error (SE) approach and 10-fold cross-validation. Consequently, nine prognostic MMPs-related DEGs were determined to be characteristic risk genes. Ultimately, the MMPs-related prognostic signature was formulated using HCC samples from the TCGA-LIHC cohort. The risk score was determined by linearly combining gene expression levels with their respective regression coefficients. The risk score for the correlation coefficient between MMPs-related prognostic signature can be calculated using the following formula: risk score = (0.0086×expression level of RNPEPL1) + (0.0009× expression level of ADAM15) + (0.5255×expression level of ADAM18) + (0.110× expression level of ADAMTS5) + (0.0126× expression level of CAD) + (0.0018×expression level of YME1L1) + (0.0025×expression level of AMZ2) + (0.0114×expression level of PSMD14) + (0.0011×expression level of COPS6) (Figure 4A, 4B).

In the entire, training and testing cohorts of the TCGA-LIHC, the heatmap displays the variation in the expression of genes between the high and low-risk groups. According to the median risk score, HCC patients were divided into high-risk and low-risk groups. The TCGA-LIHC cohort showed that the low-risk group had a considerably extended OS duration compared to the high-risk group. The results of PCA indicated a remarkable ability of the risk genes to discriminate between the two groups. The distribution of risk scores indicated a significant increase in the number of fatalities in the high-risk group as compared to the low-risk group. The results of the TCGA-LIHC cohort and the TCGA-LIHC training and testing cohorts showed that patients in the low-risk group had significantly longer OS than patients in the high-risk group (*P*<0.001). (Figure 4C, 4E) In addition, additional verification was performed in the ICGC-LIRI-JP cohort, and it was observed that the low-risk group had significantly longer OS than the high-risk group (*P*<0.001). To examine the reliability of the model, the AUC values for OS, which is a time-dependent measure, were calculated by determining the area under the ROC curves. The respective AUC values for the 1-, 3-, and 5-year OS were 0.677, 0.716, and 0.669 (Figure 4F).

### Independent predictive value of the risk model

To evaluate the individual impact of the model on HCC prognosis, univariate and multivariate Cox regression analyses were performed in both TCGA-LIHC training and testing cohorts. This analysis involved considering clinical features and the risk score. For both the training and testing cohorts, univariate Cox regression analysis demonstrated that a high-risk score was an independent predictor of adverse patient survival (*P*<0.001, HR=3.053, 95% CI: 2.071-4.502 and *P*<0.001, HR=3.316, 95% CI: 1.977-5.563, respectively (Figure 4G).

The outcome was anticipated and validated through the use of multivariate Cox regression analysis, which additionally demonstrated the use of the risk model to be an independent prognostic factor for HCC patients in both the training cohort (*P*<0.001, HR=2.690, 95% CI: 1.766-4.097) and the testing cohort (*P*=0.002, HR=2.467, 95% CI: 1.409-4.317), irrespective of other clinical factors (Figure 4H).

**Figure 5:**
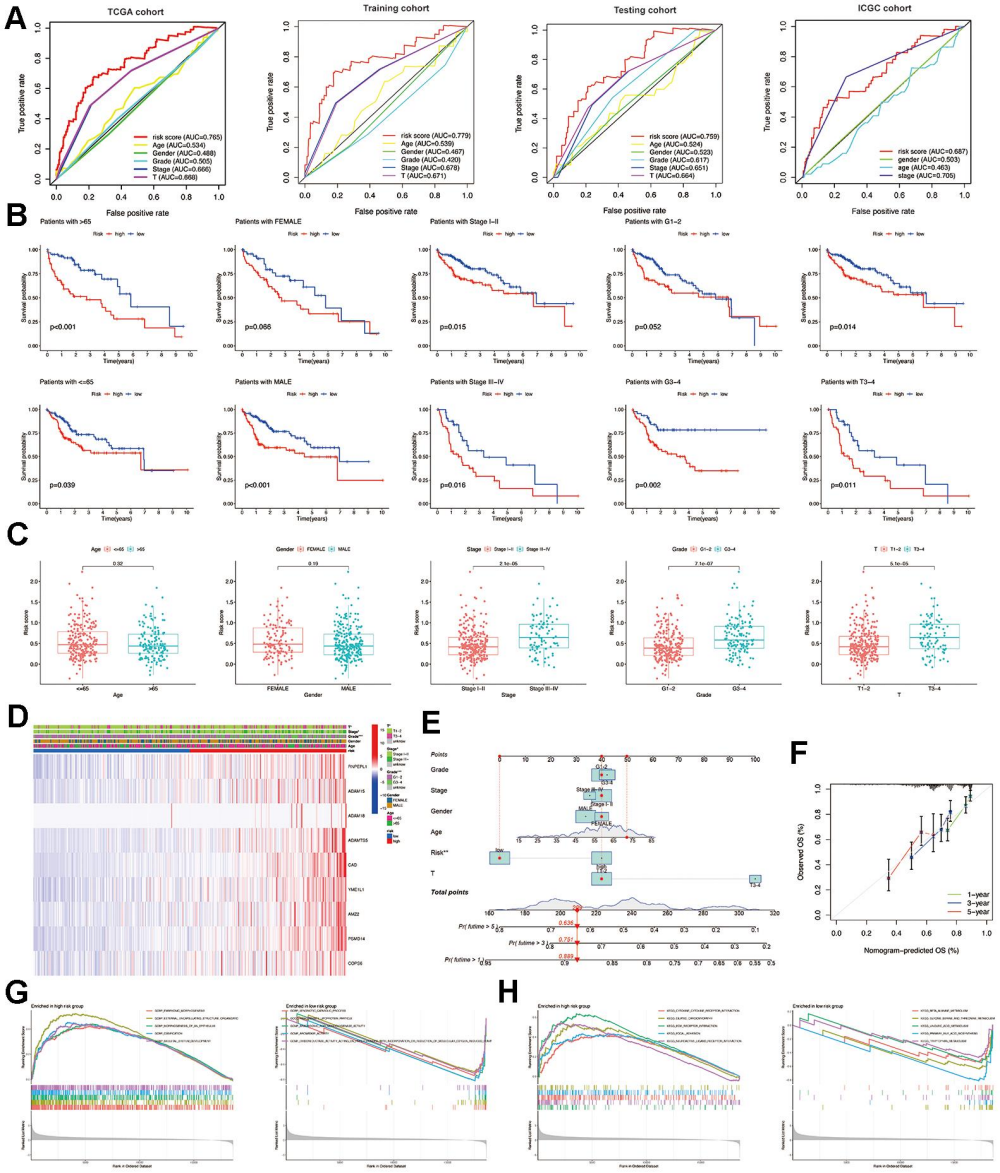
**Relationship between risk score and clinicopathological features.** (**A**) Multi-index ROC curve of the MMPs-related prognostic signature in TCGA-LIHC and ICGC-LIRI-JP cohorts. (**B**) Comparison of the predictive role of risk score for multiple clinicopathological features. (**C**) Comparison of risk scores for multiple clinicopathological features. (**D**) Summarized heatmap of the distribution of clinical characteristics and the MMPs-related prognostic signature in TCGA. (**E**) Nomogram for the quantitative prediction of 1-, 3-, and 5-year survival. (**F**) Calibration plots for predicting 1-, 3-, and 5-year survival. (**G**) The results of GO annotation for low-risk and high-risk groups by GSEA. (**H**) The KEGG annotation results of low-risk and high-risk groups by GSEA.

To further demonstrate the importance of the prognostic signature associated with MMPs, the ROC results indicate that risk scores can achieve a tremendous overall advantage compared to clinicopathological features in both the TCGA-LIHC and ICGC-LIRI-JP cohorts. The analysis involved examining the correlation between clinicopathological features and risk scores (Figure 5A). In the TCGA group, the risk scores were found to be associated with the T stage and tumor stage (*P*<0.001), whereas no association was found with age, sex, and grade (*P*>0.05) (Figure 5B). Furthermore, the associated risk score increases as the tumor stage rises (Figure 5C). The heatmap displays the variation in the expression of the prognostic signature related to MMPs across different clinical characteristics and risk categories within the TCGA-LIHC cohort (Figure 5D). Combining five prognostic factors generated a nomogram to predict the OS at 1-, 3-, and 5-year using the TCGA-LIHC data (Figure 5E). The calibration curves for the prediction of OS at 1-, 3-, and 5-year are in good agreement with the observed values (Figure 5F).

### Functional analysis of MMPs-related prognostic signature

GSEA was performed to identify the biological processes and signaling pathways in which the MMPs-related prognostic signature was enriched. The results showed that the MMPs-related prognostic signature in the high-risk group was mainly enriched in embryonic morphogenesis, external encapsulating structure organization, morphogenesis of an epithelium, ossification, and skeletal system development. In contrast, the low-risk group was significantly enriched in xenobiotic catabolic process, high-density lipoprotein particle, arachidonic acid monooxygenase activity, aromatase activity, and oxidoreductase activity acting on paired donors with incorporation or reduction of molecular oxygen reduced flavin or flavoprotein as one donor and incorporation of one atom of oxygen (Figure 5G). Results from GSEA also indicated that the MMPs-related prognostic signature was mainly enriched in pathways including ECM receptor interaction, cytokine-cytokine receptor interaction, dilated cardiomyopathy, focal adhesion, and neuroactive ligand-receptor interaction in the high-risk group. On the other hand, the group with low risk primarily had an abundance of metabolic pathways such as primary bile acid biosynthesis, beta-alanine, glycine serine and threonine, linoleic acid, and tryptophan metabolism (Figure 5H).

### Analysis of immune infiltration in high- and low-risk groups

**Figure 6:**
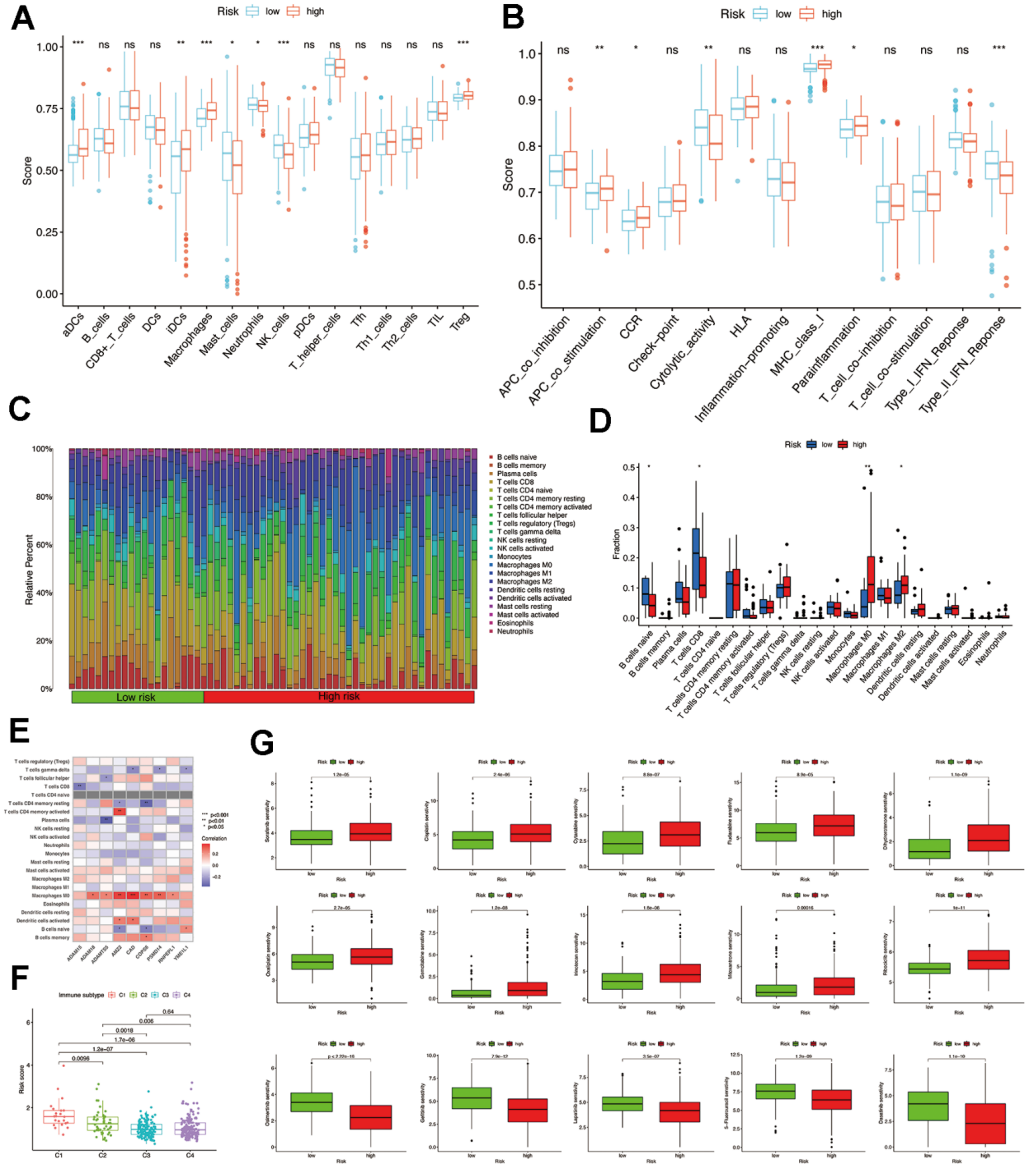
**Comparison of immune infiltration and drug sensitivity analysis.** (**A**) The scores of 16 immune cells were detected by ssGSEA analysis. (**B**) The scores of 13 immune-related functions were detected by ssGSEA analysis. (**C**, **D**) 22 types of immune cells were identified by CIBERSORT analysis. (**E**) Correlation analysis between 22 types of immune cell proportions and MMPs-related prognostic signature. (**F**) The risk score of various immune infiltration subtypes. (**G**) Sensitivity of different chemotherapeutic agents in different risk groups.

According to the analysis of ssGSEA, it was found that the group at high risk showed increased levels of expression for four immune cells, specifically aDCs, iDCs, macrophages, and Treg. The low-risk showed increased levels of three immune cells: mast cells, neutrophils, and NK cells (Figure 6A). Moreover, the high-risk group exhibited enhanced functionality of immune cells, such as APC co-stimulation, CCR, Parainflammation, and MHC class I, compared to the low-risk group (Figure 6B). Using CIBERSORT analysis, 22 immune cell profiles were detected in the low and high-risk groups. The results showed that the high-risk group had a higher percentage of M0 and M2 macrophages, while the low-risk group had larger CD8 T and naive B cells (Figure 6C, 6D). Spearman correlation analysis revealed a significant positive correlation between 9 MMPs-related prognostic genes and CD4 memory-activated T cells and M0 macrophages (Figure 6E).

Additionally, we examined the association between immune infiltrations and risk score to elucidate the role of the MMPs-related prognostic signature in the immune microenvironment. Only C1 to C4 subtypes were detected in patients with HCC, as C5 and C6 subtypes were absent. The results of our study suggest a strong association between immune-infiltrating subcategories, specifically the C1 and C2 subcategories, and elevated risk scores in the TCGA-LIHC cohort. This implies that the MMPs-related prognostic signature might influence the presence of immune infiltrates in individuals diagnosed with HCC (Figure 6F).

### Analysis of drug sensitivity

Afterward, we examined the responsiveness of various chemotherapy drugs in distinct risk categories. We observed that the IC_50_ levels of sorafenib, cytarabine, cisplatin, dihydro rotenone, fludarabine, gemcitabine, irinotecan, oxaliplatin, ribociclib, and mitoxantrone exhibited greater values within the high-risk group. While in the low-risk group, the IC_50_ values of osimertinib, lapatinib, gefitinib, dasatinib, and 5-fluorouracil were elevated (Figure 6G and Supplementary Figure 1).

### Validation of single-cell RNA-seq

**Figure 7:**
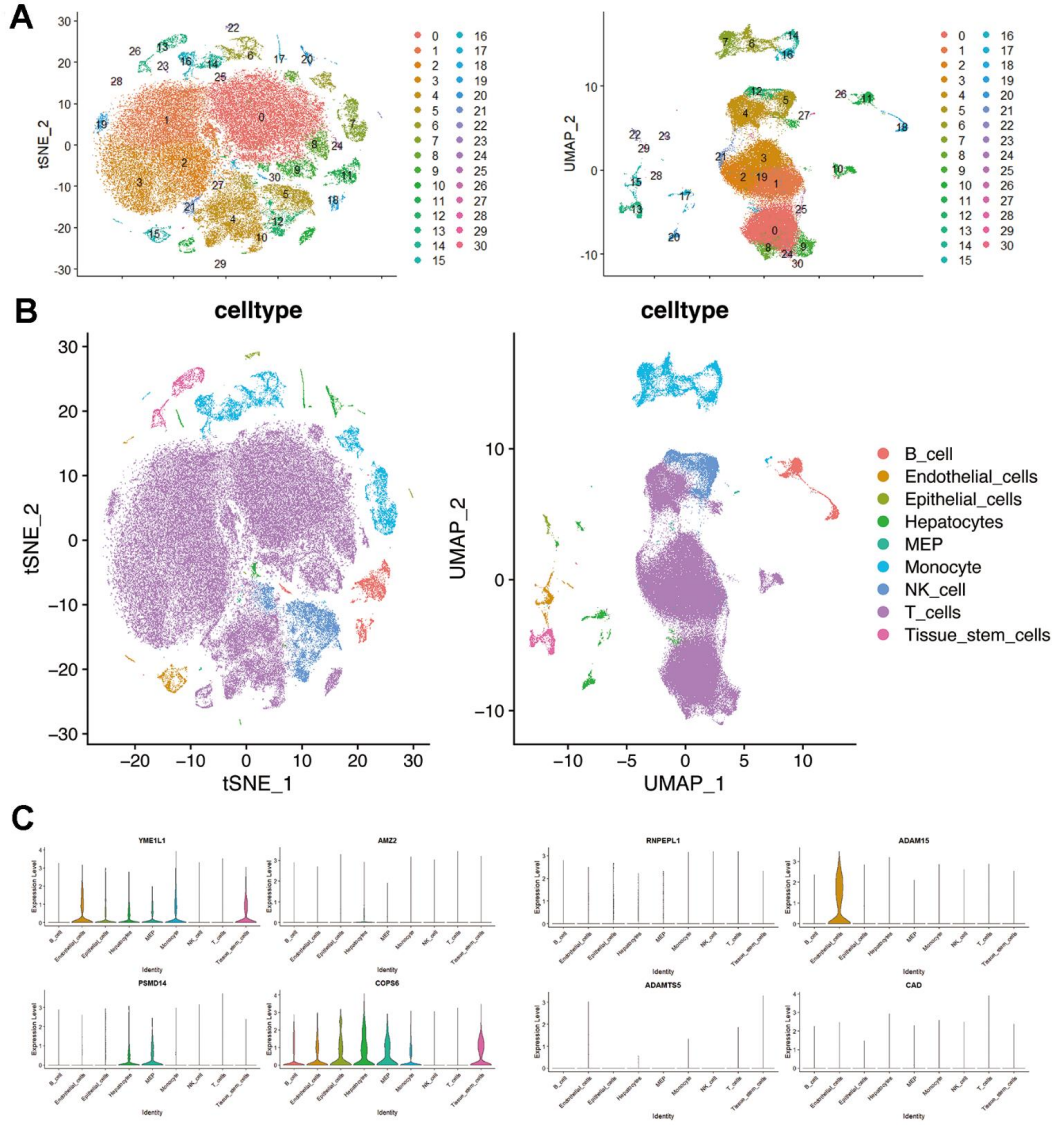
**Single-cell RNA-seq profiling in hepatocellular carcinoma.** (**A**) t-SNE and UMAP plots colored by different cell clusters. (**B**) The cell types are identified by marker genes. (**C**) Expression of MMPs-related prognostic signature in each cluster.

Seurat was employed for dimensionality reduction (Supplementary Figure 2) and clustering of the single-cell data, while SingleR was employed for cell-type annotation (Figure 7A, 7B). In the single-cell dataset, the expression of 8 genes belonging to the MMPs-related prognostic signature was identified. These genes include YME1L1, AMZ2, RNPEPL1, ADAM15, PSMD14, COPS6, ADAMTS5, and CAD. Among these genes, YME1L1 and ADAM15 were predominantly expressed in clusters of endothelial cells, while AMZ2, PSMD14, and COPS6 were predominantly expressed in clusters of hepatocytes. Additionally, YME1L1 and COPS6 were the main genes expressed in clusters of monocytes (Figure 7C, 7D).

## DISCUSSION

HCC, being a medical concern for society, continues to have a bleak outlook. It is crucial to investigate primary molecular indicators associated with the advancement and prediction of HCC, which can be employed as targets for therapy. From a pool of 203 genes related to MMPs, we obtained 13 prognostic MMPs-related DEGs. Following this, a predictive model was built utilizing LASSO Cox regression analysis, and nine MMPs-related genes were identified as a prognostic signature, showing an independent correlation with the prediction of HCC prognosis. RNPEPL1, a member of the M1 family of zinc metal peptide enzymes, consists of 12 enzymes in humans and employs Zn^2+^ as the central active site [18]. The individuals in this household participate in various activities, ranging from breaking down peptides and retrieving amino acids in overall cellular proteolysis to controlling physiological signaling sequences by breaking down peptide hormones and processing peptides displayed on class I MHC molecules [19]. Research has verified that blockers of these enzymes have demonstrated potential in managing different ailments, including high blood pressure, swelling, and even certain types of tumors [20–23]. ADAM15, a disintegrin and metalloproteinase 15, is a member of the ADAM family. It participates in numerous physiological and pathological processes by breaking down ECM and releasing membrane-bound precursors that control cell interactions and the extracellular matrix [24]. ADAM15 includes innumerable substrates, including essential molecules for cell regulation like E-cadherin and N-cadherin, TGFβ, and EGFR ligands [25]. Moreover, previous studies have shown that ADAM15 promotes the synthesis of pro-MMP-9 and enhances the breakdown of gelatin by facilitating MMP-9 mediation [26]. The presence of ADAM15 has been recorded in different types of cancerous tumors, including breast, prostate, and bladder cancer [27–29]. A recent investigation validated that ADAM15 is linked to unfavorable prognosis in patients with HCC and may be regarded as a promising biomarker for diagnosing and treatment of HCC [30]. ADAMTS, an enzyme with a thrombospondin motif known as secreted ADAM, can degrade the ECM and is involved in various biological and pathological processes, such as tissue structure, inflammation, blood vessel formation, and cancer [31]. ADAMTS5, a protease family member, is upregulated in non-small cell lung cancer (NSCLC) patients with glioblastoma and lymph node metastasis [32, 33]. Additionally, research findings indicate that ADAMTS5 may impede the advancement of HCC, presenting an opportunity for further exploration regarding ADAMTS5 as a potential prognostic marker and promising therapeutic target in HCC [34]. The YME1L1 gene, belonging to the AAA group of ATPases and an ATP-dependent metalloprotease encoded by the nuclear genome, is situated within the inner mitochondrial membrane, positioning its protease domain towards the intermembrane space [35]. The mitochondria’s entry of YME1L1 is accompanied by protein hydrolysis through the activity of the mitochondrial processing peptidase, which splits off the sequence destined for the mitochondria [36]. It is believed that YME1L1 is also implicated in the process of nuclear mitochondrial DNA insertion, and the increased expression of YME1L1 is strongly linked to ovarian cancer and the advancement of tumors [37].

Regarding the remaining genes within the set of signature genes, carbamoyl phosphate synthetase 2, aspartate transcarbamylase, and dihydroorotase (CAD) play a crucial role as they encode CAD. These enzymes are essential for pyrimidine synthesis, new pyrimidine nucleotides, protein glycosylation, and the biosynthesis of phospholipids in mammals [38, 39]. CAD is responsible for initiating the di-(UDP)-dependent glycosylation process, resulting in the production of UDP. There is a significant association between elevated CAD expression and an unfavorable prognosis of HCC [40, 41]. PSMD14, also known as Rnp11, is a metalloproteinase that includes the JAB1/MPN/Mov34 (JAMM) domain and has a Zn2^+^-ion in its active center [42]. PSMD4, functioning as a ubiquitin-degrading enzyme within the proteasome’s 19S regulatory granules, can control numerous biological processes, such as the stability of proteins, the advancement of cancer, and resistance to drugs [43]. COPS6, a member of the JAMM family, has recently been verified to facilitate tumor advancement and decrease the infiltration of CD8^+^ T-cells by suppressing the synthesis of IL-6, thus promoting tumor immune escape from cancer [44, 45].

Active participants in the development of HCC include diverse immune cells found within the tumor immune microenvironment, including macrophages, natural killer (NK) cells, DCs, tumor-associated endothelial cells (ECs), cancer-associated fibroblasts (CAFs), abnormal tumor vasculature, CD4+, and CD8+ T cells, and myeloid-derived immunosuppressive cells (MDSCs) [46]. A comprehensive examination was conducted to investigate the association between the signature related to MMPs and the infiltration of immune cells in HCC. We noticed that the high-risk population showed a higher level of immune cell infiltrations, including aDCs, iDCs, macrophages, and Treg cells. In contrast, the low-risk group showed an increased presence of activated mast cells, neutrophils, and NK cells. According to the CIBERSORT analysis, the high-risk group exhibited a higher quantity of M0 and M2 macrophages. In contrast, the low-risk group presented a higher presence of naive B cells and CD8^+^ T cells. Moreover, the vulnerable population demonstrated enhanced functionality of immune cells, such as APC co-stimulation, CCR, parainflammation, and MHC class I. This implies that the predictive function of the MMPs-associated pattern could potentially be linked to macrophages.

Tumor-related macrophages (TRMs) are an essential component of the tumor surroundings and play a role in controlling blood vessel formation, modifying the extracellular structure, promoting the growth of cancer cells, spreading, suppressing the immune system, and developing resistance to chemotherapy and immunotherapy that targets checkpoints [47]. Different functional phenotypes can be achieved by polarizing TAMs, which play a crucial role in inflammation related to tumors. The M1 macrophages, which are induced by interferon alone or in combination with lipopolysaccharide, and the M2 macrophages, which IL-4 or IL-13 induces, are the subgroups that have been extensively studied. The M1 phenotype of activated macrophages can stimulate anti-tumor immune responses by various means, such as presenting antigens to adaptive immune cells, generating pro-inflammatory cytokines, and engulfing tumor cells [48, 49]. Tumor-associated macrophages (TAMs), which are polarized toward the M2 phenotype, may also secrete the cytokine CCL22, leading to an increase in tumor invasion as well as the induction of EMT through activating Smad2/3 and Smad1/5/8 and upregulating Snail [50].

Furthermore, the secretion of CCL17 by M2 macrophages is intricately associated with tumor stemness and EMT, operating through the TGF-β1 and Wnt/β-catenin signaling pathways [51]. Moreover, studies have shown that M2 macrophages can protect tumor in a positive feedback loop by releasing HGF in HCC [52]. The current study may explain the decreased responsiveness to chemotherapeutic agents with high risk. Although macrophages are not detected in this dataset, our results show that YME1L1 and COPS6 are the significant genes expressed in monocyte clusters. Circulating monocytes are the primary source of infiltrating macrophages in tumors, so our results may suggest a potential role for YME1L1 and COPS6 in macrophage polarization in HCC.

There are several limitations to this study. First, this study uses public datasets for different patient cohorts, and the results might be heterogeneous in data processing and patient selection. Although we validated our gene signature in external datasets, prospective cohorts with more HCC patients are needed to validate our risk models. Second, the function of the MMPs-related signature in the carcinogenesis and progression of HCC needs to be further investigated.

## CONCLUSIONS

A MMPs-related prognostic signature (including RNPEPL1, ADAM15, ADAM18, ADAMTS5, CAD, YME1L1, AMZ2, PSMD14, and COPS6) was identified. These findings could potentially impact the predictability of the prognosis and treatment of HCC.

## Supplementary Material

Supplementary Figures

Supplementary Table 1
