# Supplementary material for: Identification of a novel matrix metalloproteinases-related prognostic signature in hepatocellular carcinoma
Source: Aging (Albany NY). 2024 May 16;16(10):8667–86. doi: 10.18632/aging.205832 (PMC11164509; doi:10.18632/aging.205832)
Supplement: Supplementary Figures [file aging-16-205832-s001.pdf]

SUPPLEMENTARY FIGURES

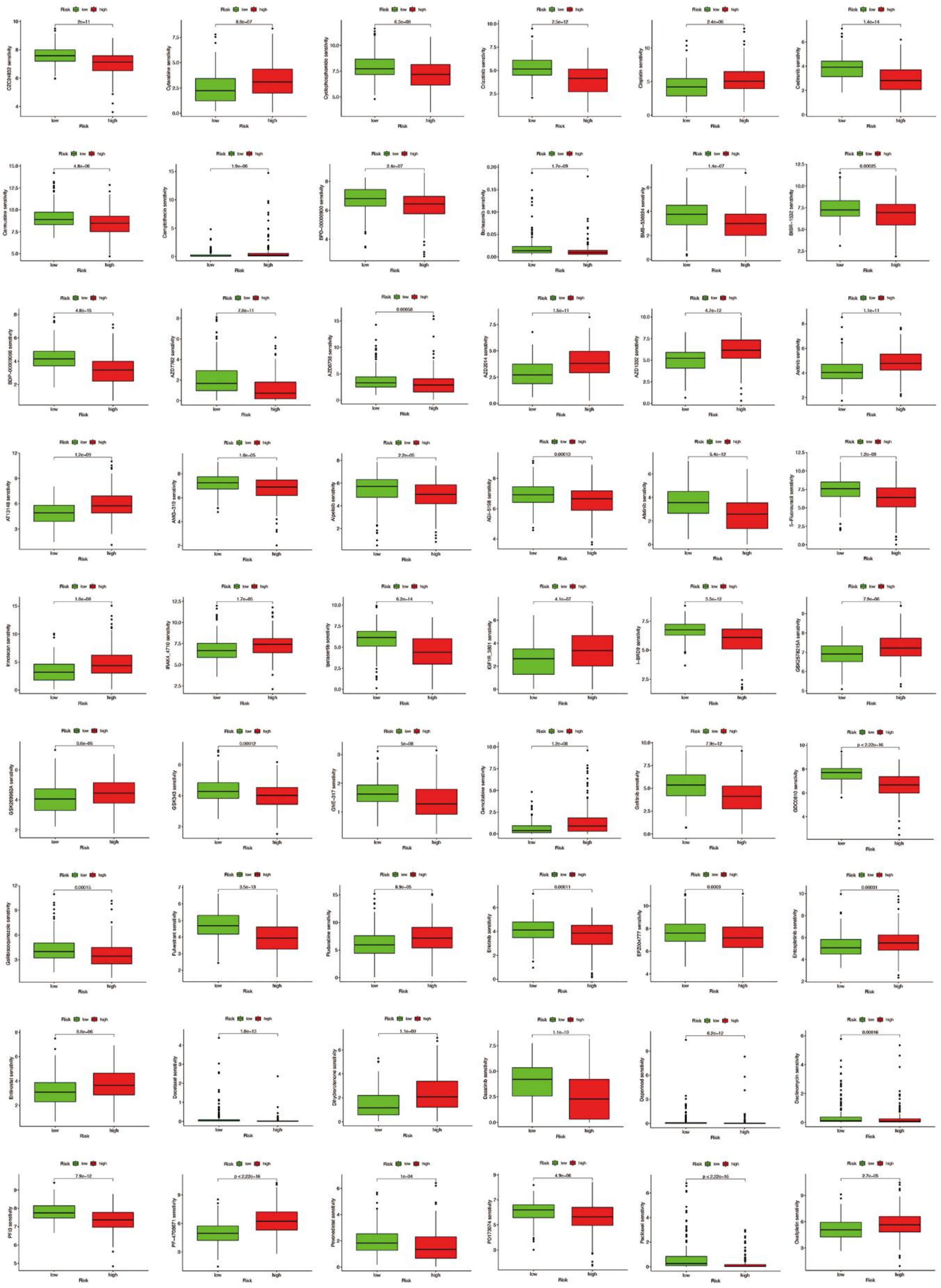

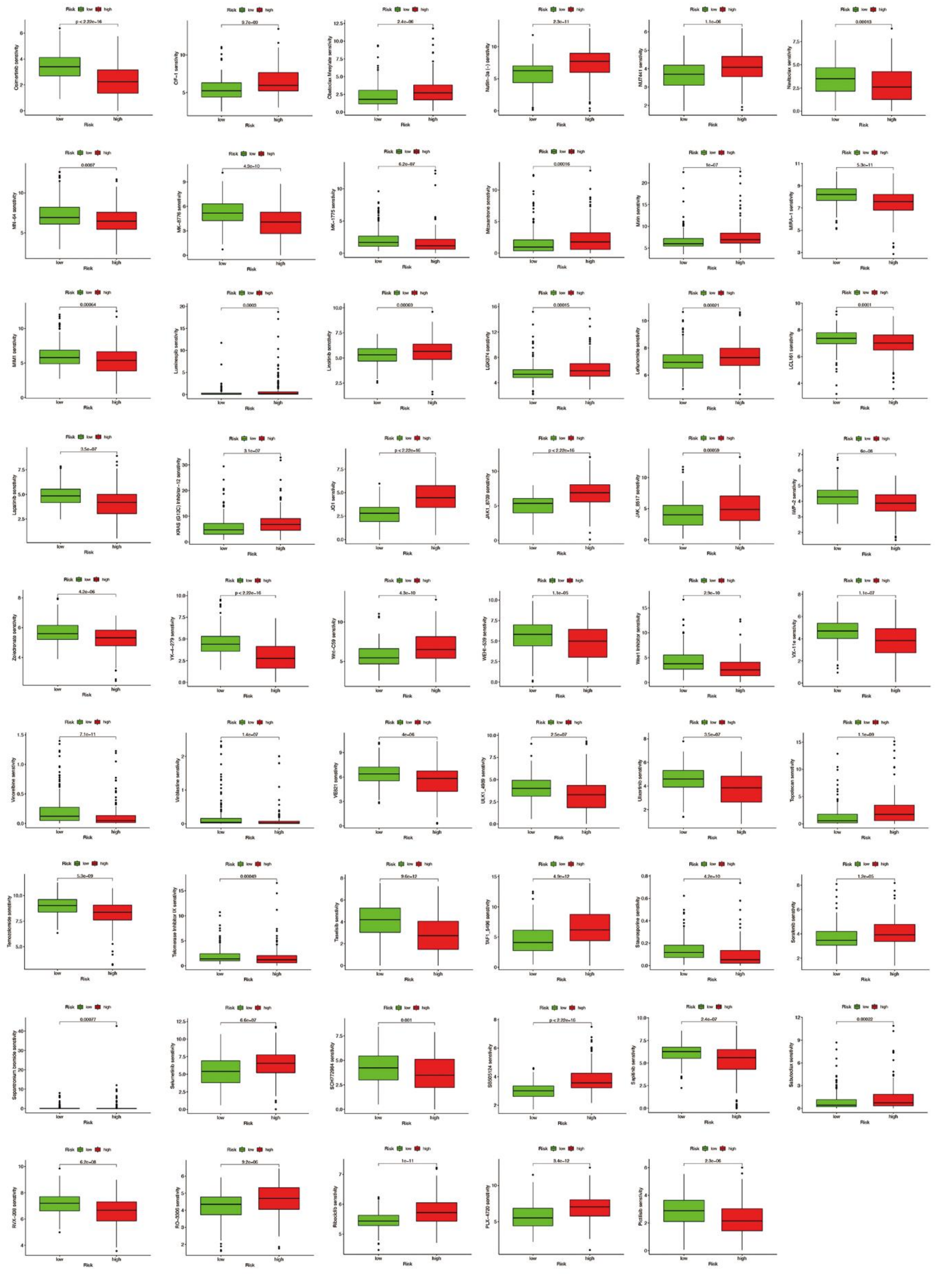

Supplementary Figure 1. Comparison of the sensitivity of various chemotherapeutic agents in distinct risk groups of the TCGA-LIHC cohort.

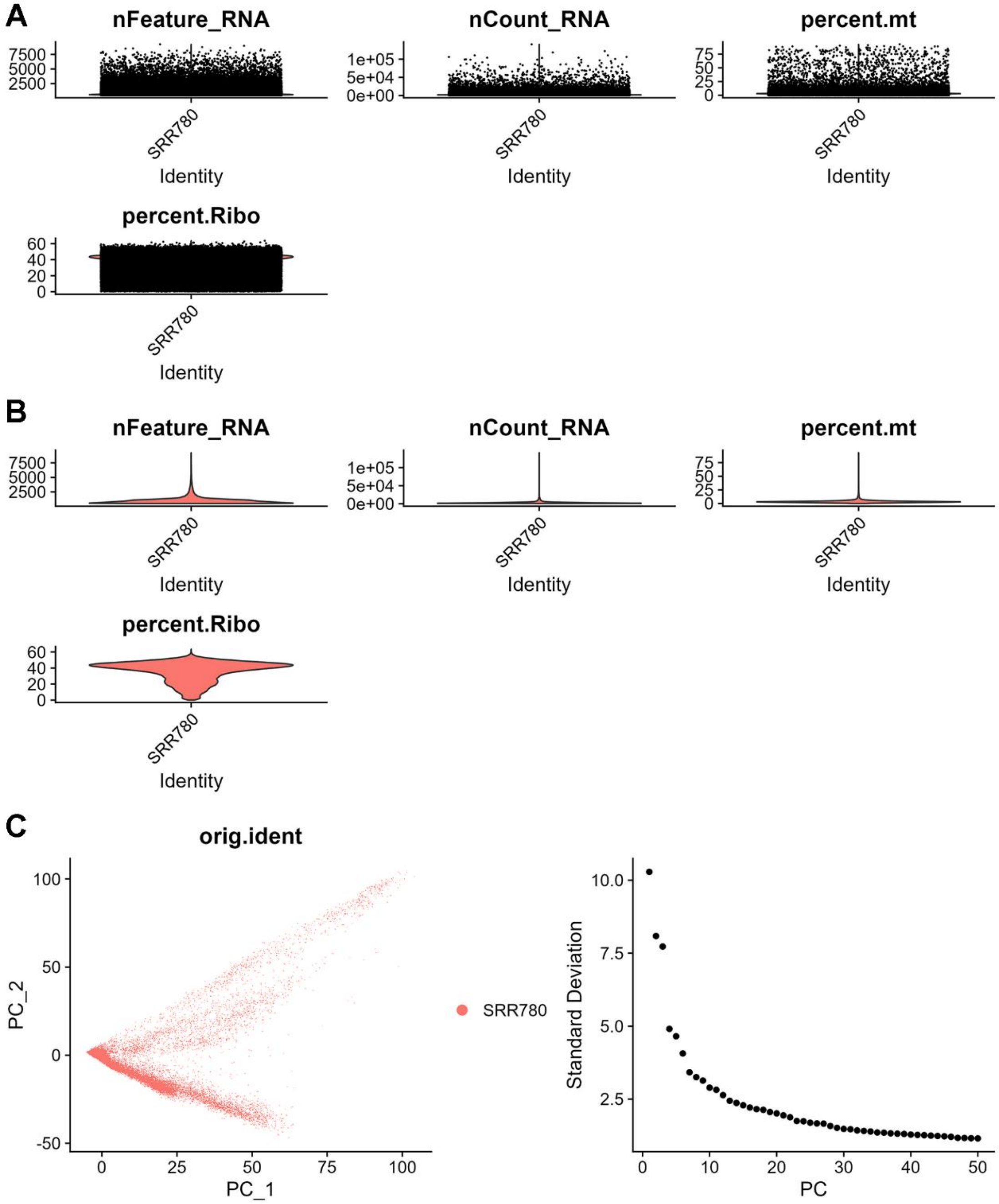

Supplementary Figure 2. Single-cell RNA-seq profiling in hepatocellular carcinoma. (**A**, **B**) The Seurat package is used to filter data from individual cells. (**C**) Utilizing dimensionality reduction and the Seurat package, single-cell data can be clustered.
